# Poster Session II - A223 ACQUIRED HEMOPHILIA A IN AN ADOLESCENT WITH ULCERATIVE COLITIS ON INFLIXIMAB: A CASE REPORT

**DOI:** 10.1093/jcag/gwaf042.223

**Published:** 2026-02-13

**Authors:** A Alammar, S Lawrence, M Belletrutti, M Smyth, L Rozka

**Affiliations:** BC Children’s Hospital, Vancouver, BC, Canada; BC Children’s Hospital, Vancouver, BC, Canada; BC Children’s Hospital, Vancouver, BC, Canada; BC Children’s Hospital, Vancouver, BC, Canada; BC Children’s Hospital, Vancouver, BC, Canada

## Abstract

**Background:**

Acquired hemophilia A (AHA) has never been reported in patients with pediatric inflammatory bowel disease (IBD).

**Aims:**

We describe a case of AHA occurring in an adolescent with ulcerative colitis treated with infliximab. We hypothesize that in rare circumstances, anti-TNF therapy can trigger factor VIII inhibitor development in patients with IBD.

**Methods:**

We reviewed the disease course of an adolescent with ulcerative colitis who developed AHA after 3 years of infliximab therapy. A focused literature review was performed to assess reports of inflammatory bowel disease and AHA.

**Results:**

The patient presented at 14 years of age with a 3-month history of bloody diarrhea and raised serum and fecal biomarkers. His colonoscopy showed a Mayo 2 pancolitis. After a course of oral prednisone, he was commenced on infliximab monotherapy in September 2022. He achieved sustained clinical remission with normal serum and fecal biomarkers. Repeat scope in July 2024 showed endoscopic and histologic remission. Intestinal ultrasound in July 2025 showed transmural remission. In July 2025, he presented with spontaneous bruising and palpable purpura on his legs. Laboratory investigations revealed a prolonged aPTT, factor VIII activity <0.01 IU/mL, and a high-titer inhibitor (49 BU), consistent with AHA. Malignancy, infection, and systemic autoimmune disease were excluded. He was treated with recombinant activated factor VII, emicizumab, high dose prednisone plus rituximab. Infliximab was discontinued. Over four weeks he had no further bruising episodes, factor VIII levels improved (to 0.16 U/mL) and inhibitor titer decreased (to 1 BU). He remains on emicizumab, continues to wean prednisone, and will commence an alternate pathway advanced therapy in the next month. He remains in clinical, serum and fecal biomarker remission with transmural remission on intestinal ultrasound. A Literature review confirmed four adalimumab-associated AHA cases. Infliximab has not previously been associated with AHA. Moreover, there have been no prior pediatric IBD reports of AHA.

**Conclusions:**

This case represents the first pediatric report of infliximab-associated AHA in ulcerative colitis. It expands the spectrum of paradoxical autoimmune complications of anti-TNF therapy in IBD. Gastroenterologists should suspect AHA in IBD patients presenting with unexplained bleeding and prolonged aPTT, even in the context of disease remission. Early recognition and multidisciplinary management are essential to avoid morbidity and guide safe transition to alternate pathway advanced IBD therapies.

**Funding Agencies:**

None

